# Impaired behavioral inhibitory control of self-injury cues between adolescents with depression with self-injury behavior and those without during a two-choice oddball task: an event-related potential study

**DOI:** 10.3389/fpsyt.2023.1165210

**Published:** 2023-06-12

**Authors:** Lingli Ma, Dong-Dong Zhou, Lin Zhao, Jinhui Hu, Xinyu Peng, Zhenghao Jiang, Xiaoqing He, Wo Wang, Su Hong, Li Kuang

**Affiliations:** ^1^Department of Psychiatry, The First Affiliated Hospital of Chongqing Medical University, Chongqing, China; ^2^Mental Health Centre, University-Town Hospital of Chongqing Medical University, Chongqing, China

**Keywords:** event-related potential, behavior inhibitory control, P3, non-suicidal self-injury, suicidality, time-frequency analysis

## Abstract

**Background:**

This study aimed to objectively evaluate the severity of impulsivity [behavior inhibitory control (BIC) impairment] among adolescents with depression. In particular, those involved in non-suicidal self-injury (NSSI) behaviors, compared with those engaged in suicidal behaviors and adolescents without any self-injury behavior, using event-related potentials (ERPs) and event-related spectral perturbation (ERSP) within the two-choice oddball paradigm.

**Methods:**

Participants with a current diagnosis of major depressive disorder (MDD) engaged in repetitive NSSI for five or more days in the past year (*n* = 53) or having a history of at least one prior complete suicidal behavior (*n* = 31) were recruited in the self-injury group. Those without self-injury behavior were recruited in the MDD group (*n* = 40). They completed self-report scales and a computer-based two-choice oddball paradigm during which a continuous electroencephalogram was recorded. The difference waves in P3d were derived from the deviant minus standard wave, and the target index was the difference between the two conditions. We focused on latency and amplitude, and time-frequency analyses were conducted in addition to the conventional index.

**Results:**

Participants with self-injury, compared to those with depression but without self-injury, exhibited specific deficits in BIC impairment, showing a significantly larger amplitude. Specifically, the NSSI group showed the highest value in amplitude and theta power, and suicidal behavior showed a high value in amplitude but the lowest value in theta power. These results may potentially predict the onset of suicide following repetitive NSSI.

**Conclusion:**

These findings contribute to substantial progress in exploring neuro-electrophysiological evidence of self-injury behaviors. Furthermore, the difference between the NSSI and suicide groups might be the direction of prediction of suicidality.

## 1. Introduction

The global age-standardized suicide rate was 9.0 per 100,000 people with a sharp increase among young people aged between 10 and 20 years, according to the database published by WHO in 2022. The Chinese national suicide rates have decreased at a slow pace since 2006 (1). However, regional suicide mortality changed with a downward trend before 2009, followed by an upward trend as reported, based on reports (2). Clinical research has shown that cross-cutting constructs, such as affective and behavioral disorders, may be stronger predictors of suicidal events than single risk factors (e.g., past suicide attempts or psychiatric diagnoses) (3). Even if self-injury behaviors are not lethal, research also emphasized them (4). However, very few studies of suicidality focus on the affective combined with self-injury behaviors.

Self-injury behaviors, ranging from non-suicidal self-injury (NSSI) to suicidal behaviors, are a broad class of actions aimed at directly and deliberately injuring oneself (5). Although it is generally accepted that suicidal ideation confers a risk for later behaviors, extensive epidemiological and meta-analytical studies have found that it does not meaningfully differentiate individuals with a high risk of suicide attempts (6, 7). Theoretical models of suicide suggest that the factors for the development of suicidal ideation are distinct from those involved in the transition from thoughts to attempts (8–10). Among the meta-analytical studies, NSSI is one of the most robust predictors of future suicidal behaviors, even though it is defined as self-harm without the intent to die (11–15). How does NSSI increase the risk of suicidality? Notably, experiences of NSSI change the feelings and sensations of pain or such “risky behaviors” to provide emotional relief or balance more significant emotional dysregulation (16). In turn, such changes result in severe self-injury during future mental stress until the individual can no longer cope with it, and other suicidal behavior results (9, 11).

With immature and unstable impulse control, adolescents are a high-risk group for repetitive NSSI in the context of emotion dysregulation. A meta-analysis found that adolescents' aggregate prevalence rates of NSSI worldwide were 22.0% over their lifetime and 23.2% during 12 months (17). Simultaneously, a longitudinal study (18) found that self-injury frequency significantly predicts suicidal behavior among adolescents with NSSI. This effect is more pronounced among adolescents with depression (19, 20). Given that these two risk factors share a high common rate among the youth, we focused on adolescents with depression who engage in repetitive self-injury behaviors. This was to investigate whether there is a behavioral development spectrum from NSSI to suicide.

Therefore, it is necessary to highlight the common risk factors of NSSI and suicidal behaviors. Previous research (7, 10, 21, 22) indicated that impulsivity could be associated with the onset of a subsequent NSSI and suicide. In the behavioral dimension, impulsivity is regarded as maladaptive behavior and mainly manifested as deficits in behavior inhibitory control (BIC) (23). From the perspective of biobehavioral dispositions and biological behavior tendency, weak inhibitory control is an independent risk factor of suicidal behavior tendency (24). Its evidence comes from the study of impulsive–aggressive traits, one of the most promising endophenotypes (25), and manifested impairment of BIC showed a strong predictive relationship with suicidal behavior (26). Furthermore, laboratory tasks found that repetitive NSSI is associated with neurocognitive impulsivity (27), and strengthening inhibitory control would help reduce self-injury behaviors (including NSSI and suicidal behaviors) (28). Both from the motivation of self-injury addiction (28) and executive function (29), the impairment of BIC is positively correlated with the severity of NSSI (30). To further clarify the formation and development mechanism of NSSI and the subsequent suicidal behavior, it will be significant to focus research attention on the characteristics of BIC impairment. However, a few studies have focused on the BIC impairment difference between the two behaviors, which can be objectively measured. Thus, an essential next step in revealing the development of self-injury behaviors is to examine BIC impairment levels in both subjective and objective manners among depressive adolescents with repetitive NSSI and suicidal behaviors.

Benefitting from their excellent temporal resolution and goal-oriented evoked responses, event-related potentials (ERP) allow a high degree of target cognitive processes, such as inhibitory control (31). Although underutilized in studies of self-injury, ERP has been widely used in assessing cognitive process impairment in depression (32) and behavior using non-traditional approaches (33). Researchers frequently used the go or no-go task to investigate inhibitory control. Participants are typically asked to generate a motor response to the go trial and no motor response to the no-go trial. However, differences in motor response are likely to contaminate the inhibitory control effect because higher cognitive processes are particularly susceptible to motor potentials (34, 35). Therefore, the present study used a two-choice oddball task, in which participants were required to respond to both standard and deviant stimuli by pressing different keys as quickly as possible. Two ERP components have been suggested to reflect BIC activity, N2 and P3. N2, the negative component ~200 ms after stimulation, operates as a detector of conflicts but not real inhibitory braking. P3, ~300–500 ms after stimulation, is a slow centro-parietal positive component. It is closely related to motor inhibition in the premotor cortex (23). As reported, higher motor preparation represents more inhibition needed to withhold and more effort to respond correctly; consequently, a larger P3 is induced (36).

The present study randomized the onset sequence of standard and deviant stimuli for each participant. Compared with the 25% occurrence of deviant stimuli, standard stimuli were presented much more frequently to induce prepotent responses. Participants were required to inhibit the prepotent response and change to correct responses when they encountered deviant stimuli. To avoid overlap with adjacent ERP components, we used the different waves of the standard and deviant stimuli to isolate the components of interest. Subsequently, the difference waves to neutral cues minus self-injury cues were used as the target index. In addition to ERP, time-frequency decompositions were conducted for more BIC impairment details. Correlational studies have underlined the importance and potential of time-frequency-based indices in prospectively predicting adverse outcomes (37).

Altogether, the present study's primary goal was to explore the developmental spectrum from NSSI to suicidal behaviors among adolescents with depression, depending on the BIC impairment level. Thus, we chose BIC's classic experimental paradigms to objectively evaluate the impulsive difference between neutral and self-injury cues through ERP or event-related spectral perturbation (ERSP). We hypothesized that these indicators would differentiate individuals with repetitive self-injury behaviors and that the difference in NSSI and suicidal behaviors would reflect the developmental spectrum. Due to limited knowledge about the severity of BIC impairment among adolescents with depression, we also collected the ERPs of adolescents with depression without any self-injury behavior as a control group. Finally, we employed time-frequency analyses to measure oscillatory neural activity in the theta frequency band. Although the present study focused on objective behavioral indicators, we utilized a correlation analysis to examine whether they share consistency.

## 2. Methods and materials

### 2.1. Participants

The participants of this study were 124 adolescents aged between 12 and 17 years from the outpatient department of The First Affiliated Hospital of Chongqing Medical University. All the participants were under the supervision of their parents. Guardians and participants themselves both had signed informed consent for this study. Participants with a current diagnosis of major depressive disorder (MDD) and a history of at least one prior complete suicide behavior history (*n* = 31) in the last year were eligible for the MDD+SA group, all of whom reported a history of prior NSSI. Participants with a current diagnosis of MDD and engaged in repetitive NSSI on five or more days (*n* = 53) in the past year were eligible for the MDD+NSSI group. Participants with a current MDD diagnosis and without self-injury behavior (*n* = 40) were included in the MDD group. Healthy controls (HC group) were age-matched adolescents recruited from the same community via advertisements (*n* = 30). The exclusion criteria for all groups comprised: (1) current diagnosis of other mental illnesses such as schizophrenia, bipolar disorder, or substance dependence; (2) previous brain organic mental disorders; (3) neurodevelopmental disorders; (4) other chronic or severe physical conditions. The demographic information of the four groups is presented in Table 1.

**Table 1:** Demographic and scale assessment.

**Measure**	**HC group (*n* = 30)**	**MDD group (*n* = 40)**	**MDD + NSSI group (*n* = 53)**	**MDD + SA group (*n* = 31)**	**Test statistics**
**Demographics**					
Age	15.2 ± 1.65	15.3 ± 1.47	14.4 ± 1.57	14.6 ± 1.43	*F*_(4, 154)_ = 2.055 *p* = 0.109
Female	18	23	37	24	
	60%	57%	69%	77%	χ^2^ = 3.923 *p* = 0.270
**Scale assessment**					
PHQ-9		19 ± 4.994	19.47 ± 4.539	20.25 ± 3.640	*F*_(3, 124)_ = 0.481 *p* = 0.619
Beck_lately		0.7 ± 0.622	0.57 ± 0.607	1.2 ± 0.554	*F*_(3, 124)_ = 7.875 *p* = 0.001
Beck_lifetime		1.05 ± 0.850	0.97 ± 0.825	1.79 ± 0.358	*F*_(3, 124)_ = 8.612 *p* < 0.001
BIS_MI		27.24 ± 6.247	27.34 ± 6.941	27.25 ± 5.902	*F*_(3, 124)_ = 0.002 *p* = 0.998
BIS_CI		27.68 ± 6.196	25.85 ± 7.045	25.20 ± 5.935	*F*_(3, 124)_ = 0.924 *p* = 0.401
BIS_NPI		22.84 ± 5.242	21.63 ± 6.507	22.05 ± 7.265	*F*_(3, 124)_ = 0.280 *p* = 0.757

### 2.2. Measures

#### 2.2.1. Diagnosis of major depressive disorder

The MINI-International Neuropsychiatric Interview (M.I.N.I. KID 5.0) was used to assess the current diagnosis of major depressive disorder (38) by two well-trained psychiatrists.

#### 2.2.2. Non-suicidal self-injury and suicide behaviors history and characteristics

The Ottawa Self-injury Inventory was used to assess the lifetime and latest year NSSI history and its characteristics (39). Negative emotions induced all their NSSI behaviors, and the primary purpose of the behaviors was to aid emotional dysregulation. The Columbia Suicide Severity Rating Scale was used to assess lifetime and latest month suicide behavior history (40). Those with at least one suicidal behavior in recent years were recruited in the MDD+SA group. The Beck Scale for Suicide Ideation (BSSI) was used to assess suicidal ideation (41).

#### 2.2.3. Impulsivity symptoms (self-report)

The Barratt Impulsiveness Scale (BIS) was used to assess behavior impulsivity (42). With the three dimensions of impulsivity, a higher score of motor impulsiveness and a lower score of non-planning impulsiveness will indicate impairment of behavior inhibitory control (43).

#### 2.2.4. Behavior inhibitory control impairment (event-related potential)

Participants were required to complete a visually evoked task of the two-choice oddball paradigm for ERP use. This study chose Cz as the electrode of interest, referring to a previous study (23, 44) and the characteristics of P3 potential.

**Figure 1:**
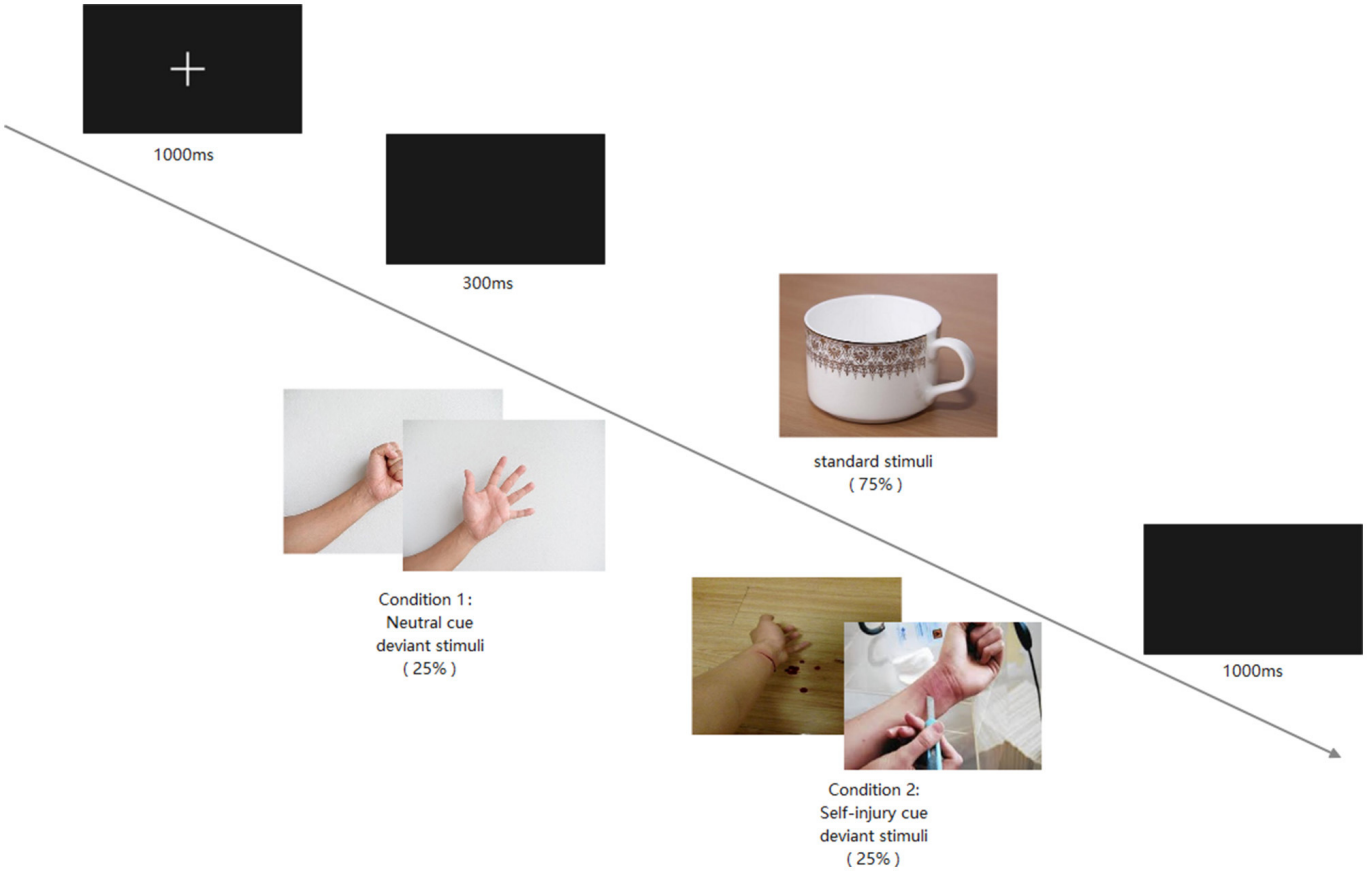
The processor of two-choice odd-ball paradigm.

The experimental environment was controlled for temperature, brightness, and noise. E-prime 3.0 was used to present the stimuli and experimental instructions. Furthermore, the two-choice oddball paradigm procedure is illustrated in Figure 1. The paradigm included two conditions: the deviant stimuli of one condition were neutral pictures, and the other was self-injury pictures. In both conditions, the standard stimuli were the same cup picture, and the standard and deviant stimuli frequencies were 75 and 25%, respectively. Participants were asked to press the “1” key in response to the standard stimuli and the “2” key in response to the deviant stimuli. Each participant was required to respond quickly and achieve at least 80% accuracy.

A Neuroscan Quick cap with 64 scalp sites was employed to record brain electrical activities, and the EEGLAB toolbox in MATLAB 2013b was used for offline analysis. The sampling rate was 1,000 Hz, and the data were resampled to 500 Hz for analysis. The impedance of each electrode was controlled below 5 kΩ, and the M1 and M2 electrodes were chosen as offline references. The offline bandpass was 0.1–30 Hz, and all EEG data were epoched to 3,000 ms, including 1,000 ms of the pre-stimuli and 2,000 ms of the post-stimuli period. Only epochs of correct responses were used for the subsequent analysis. After artifact components by independent component analysis, epochs were overlapped and averaged for each condition. The pre-stimulus period was used as a baseline to correct the post-stimulus period.

#### 2.2.5. Statistical analysis

Questionnaire data and the ERP index of BIC were analyzed using repeated-measures ANOVA. Spearman rank correlation was used to analyze the scale results and ERP index. *Post hoc* analyses were performed between the self-reported results and ERP index (latency and amplitude of P3) for the four groups (HC, MDD, MDD+NSSI, and MDD+SA). Bonferroni correction was used for multiple comparisons.

#### 2.2.6. Ethics statement

The ethics committee of the University Town Hospital of Chongqing Medical University approved all experimental procedures.

## 3. Results

The four groups were almost equally distributed by sex (χ^2^ = 3.923, *p* = 0.270) and age [*F*_(4, 154)_ = 2.055, *p* = 0.109]. Most of them were from middle school and were in the custody of their parents.

### 3.1. Self-reported results

The PHQ-9 was used to assess depressive symptoms. The scores of the three groups (MDD, MDD+NSSI, and MDD+SA) were not significantly different [mean (SD) score,19.00 (4.994) vs. 19.47 (4.539) vs. 20.25 (3.640); *F*_(3, 124)_ = 0.481, *p* = 0.619]. Meanwhile, the assessment result of self-reported impulsivity (BIS, including the three dimensions) also showed no significant difference among the three groups [BIS_MI, *F*_(3, 124)_ = 0.002, *p* = 0.998; BIS_CI, *F*_(3, 124)_ = 0.924, *p* = 0.401; BIS_NPI, *F*_(3, 124)_ = 0.280, *p* = 0.757]. Only the assessment result of suicide ideation (BSSI, including the latest week and lifetime) showed significant differences between groups [latest week, *F*_(3, 124)_ = 7.875, *p* = 0.001; lifetime, *F*_(3, 124)_ = 8.612, *p* < 0.001]; the details of the scales are presented in Table 1. *Post hoc* tests showed that the MDD+SA group was significantly higher both in the lifetime and the latest week of suicide ideation, but no significant difference between MDD and MDD+NSSI groups (Bonferroni-adjusted, *p* < 0.05); details are presented in Table 2.

**Table 2:** BSI scale comparison between groups.

**Dependent variable**		**(I) group**	**(J) group**	**Mean difference (I-J)**	**Std. error**	**Sig**.
Beck_lately	Bonferroni	MDD	NSSI	0.130	0.132	0.981
			SA	−0.497^*^	0.169	0.012
		NSSI	MDD	−0.130	0.132	0.981
			SA	−0.627^*^	0.159	0.000
		SA	MDD	0.497^*^	0.169	0.012
			NSSI	0.627^*^	0.159	0.000
Beck_mostly	Bonferroni	MDD	NSSI	0.082	0.169	1.000
			SA	−0.739^*^	0.216	0.003
		NSSI	MDD	−0.082	0.169	1.000
			SA	−0.821^*^	0.203	0.000
		SA	MDD	0.739^*^	0.216	0.003
			NSSI	0.821^*^	0.203	0.000

* *p* < 0.05.

### 3.2. Two-choice oddball task event-related potentials

As we required the participants to achieve 80% accuracy and respond as quickly as possible, the behavioral index (accuracy and latency for press response) was subject to intervention. Thus, the present study does not discuss behavioral indexes.

**Figure 2:**
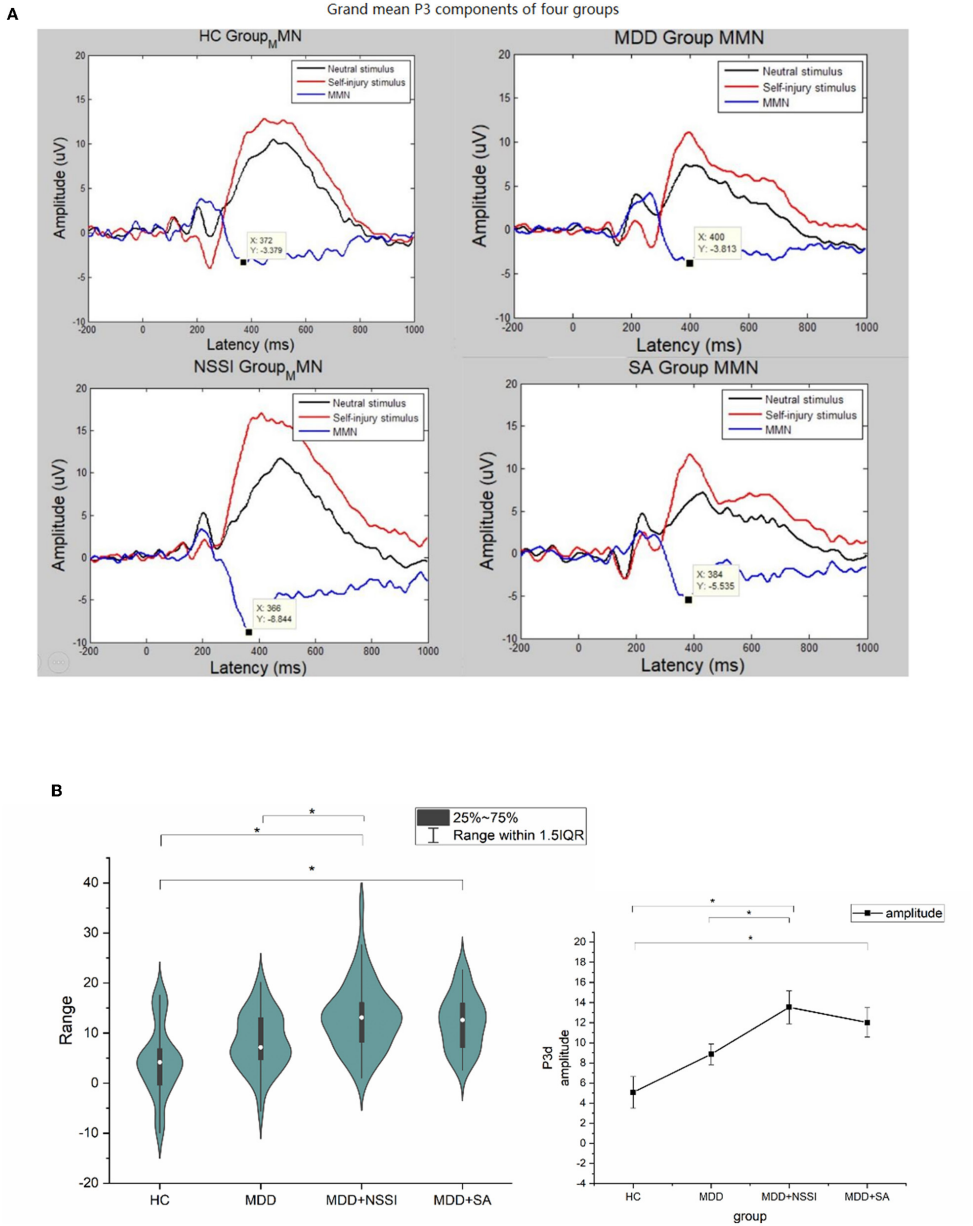
Grand mean ERPs at the group level **(A)** and P3d mean plots **(B)** on Cz channel, In order to avoid visual interference, P3d (blue curve) was changed to mirror negative curve (neutral - NSSI), P3d value remained unchanged in statistical analysis, *p < 0.05.

Regarding P3 amplitude and latency, we focused on the difference between the two cues, with the result of P3d (self-injury cue–neutral cue) as the target. We conducted two four groups (HC, MDD, MDD+NSSI, MDD+SA) × Channel mixed ANOVA of one variance with the P3d amplitude or latency serving as the dependent variable. As presented in Table 3A, the main effect of group [*F*_(4, 154)_ = 8.857, *p* < 0.001, $\eta_{\text{p}}^{2}$ = 0.21] and the main and interaction [*F*_(4, 154)_ = 8.857, *p* < 0.001, $\eta_{\text{p}}^{2}$ = 0.78] effects both showed significance. *Post hoc* tests showed that there was no significant difference in FC, and FCZ channels, but the parietal lobe channel showed a significant difference, indicating that the target channel is Cz. *Post hoc* tests indicated that the P3d amplitudes in the MDD + NSSI (Bonferroni-adjusted *p* < 0.001) and MDD + SA (Bonferroni-adjusted *p* = 0.003) groups were significantly larger than those in HC. Furthermore, the P3d amplitude in the MDD + NSSI group was considerably more extensive than in the MDD group (Bonferroni-adjusted *p* = 0.024). However, the main effect of group and interaction effects were not significant in the P3d latencies (Table 3B). Grand mean ERPs at the group level and P3d mean plots are presented in Figure 2.

**Table 3A:** One-Way ANOVA of P3d amplitude, group1 represent health control (HC) group; group 2 represent major depressive disorder (MDD) group; group 3 represent non-suicidal self-injury (MDD+NSSI) group; group 4 represent suicide behavior (MDD+SA) group.

**Dependent variable**		**(I) group**	**(J) group**	**Mean difference (I-J)**	**Std. error**	**Sig**.
CZ	Bonferroni	1	2	−3.78734	1.66814	0.153
			3	−8.45351^*^	1.78888	0.000
			4	−6.94707^*^	1.91349	0.003
		2	1	3.78734	1.66814	0.153
			3	−4.66617^*^	1.58254	0.024
			4	−3.15973	1.72214	0.418
		3	1	8.45351^*^	1.78888	0.000
			2	4.66617^*^	1.58254	0.024
			4	1.50644	1.83934	1.000
		4	1	6.94707^*^	1.91349	0.003
			2	3.15973	1.72214	0.418
			3	CPZ 1.50644	1.83934	1.000
CPZ	Bonferroni	1	2	CPZ 1.62599	1.73776	1.000
			3	CPZ 7.36322^*^	1.86354	0.001
			4	CPZ 5.71856^*^	1.99335	0.030
		2	1	1.62599	1.73776	1.000
			3	CPZ 5.73723^*^	1.64858	0.005
			4	CPZ 4.09257	1.79401	0.148
		3	1	7.36322^*^	1.86354	0.001
			2	5.73723^*^	1.64858	0.005
			4	1.64467	1.91610	1.000
		4	1	5.71856^*^	1.99335	0.030
			2	4.09257	1.79401	0.148
			3	CPZ 1.64467	1.91610	1.000

* *p* < 0.05.

**Table 3B:** One-Way ANOVA of P3d latency.

		**Sum of squares**	***df***	**Mean square**	***F***	**Sig**.
FC	Between groups	17,511.935	3	5,837.312	1.256	0.294
	Within groups	474,145.725	102	4,648.488		
	Total	491,657.660	105			
FCZ	Between groups	17,908.113	3	5,969.371	1.366	0.258
	Within groups	445,885.887	102	4,371.430		
	Total	463,794.000	105			
CZ	Between groups	19,140.205	3	6,380.068	1.334	0.268
	Within groups	487,908.700	102	4,783.419		
	Total	507,048.906	105			
CPZ	Between groups	32,166.524	3	10,722.175	2.166	0.097
	Within groups	504,943.854	102	4,950.430		
	Total	537,110.377	105			
PZ	Between groups	23,714.057	3	7,904.686	1.666	0.179
	Within groups	483,934.434	102	4,744.455		
	Total	507,648.491	105			

### 3.3. Correlation analysis

**Figure 3:**
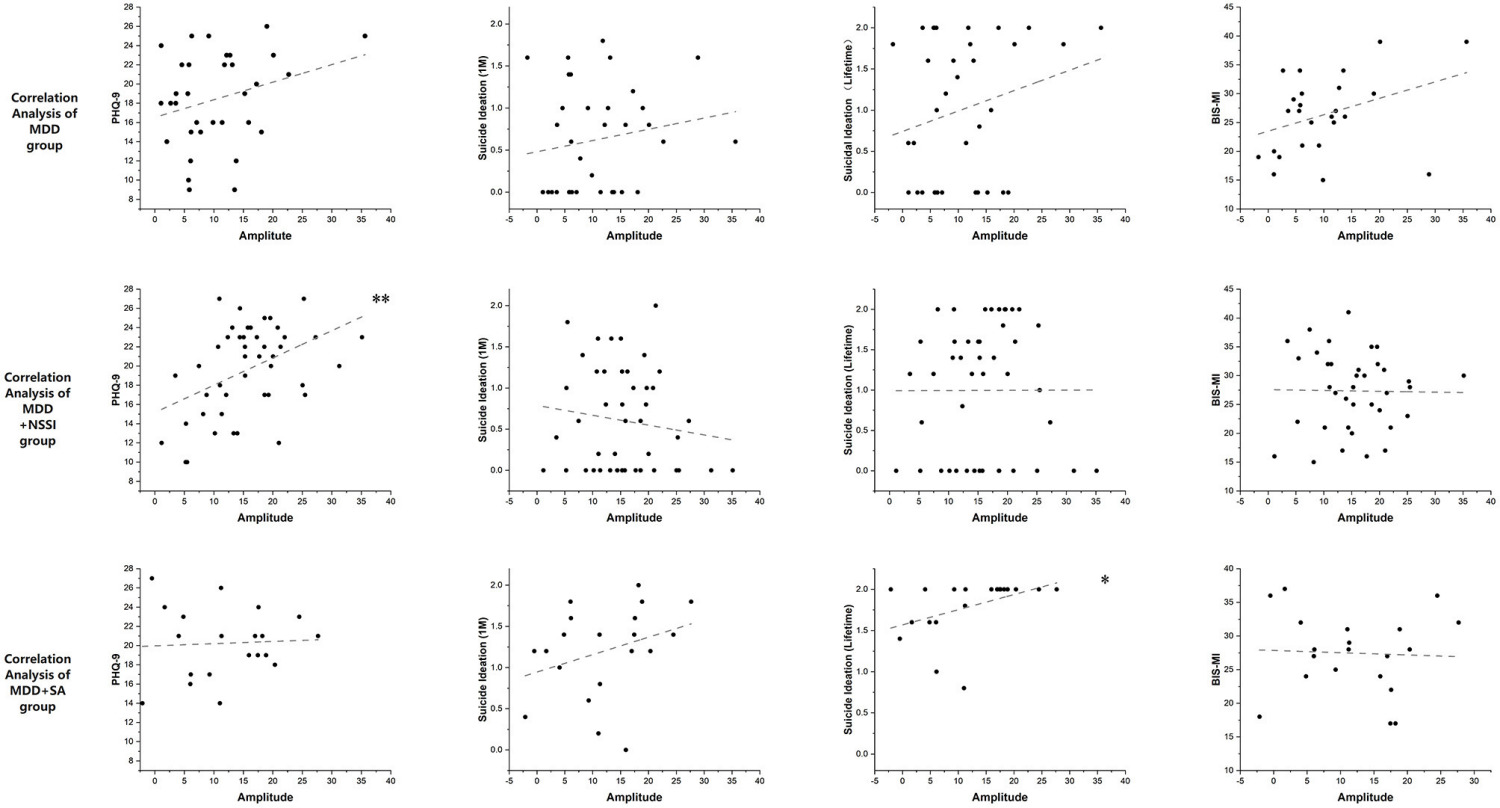
The scatter diagrams of the correlation analysis between P3d amplitude and self-reported scales, *p < 0.05, **p < 0.01.

For the three depressive groups (MDD, MDD + NSSI, and MDD + SA), correlation analysis was performed between the self-reported results (PHQ-9, BSSI, and BIS) and P3d amplitude. Interestingly, the amplitude in the MDD + NSSI and MDD + SA groups showed a significant correlation with some of the self-reported results, even with a smaller sample size, because some participants required a non-real name for their scale results. As shown in Table 4, the P3d amplitude was positively correlated with the PHQ-9 score (*r* = 0.403, *p* = 0.006, CI = 0.01) in the MDD + NSSI group and was positively correlated with the BSI (lifetime) score in the MDD + SA group (*r* = 0.559, *p* = 0.01, CI = 0.05). The scatter diagrams are shown in Figure 3.

**Table 4:** The correlation analysis between P3d amplitude and self-reported results.

			**Amplitude**
MDD group Spearman's rho	PHQ9	Correlation coefficient	0.215
		Sig. (2-tailed)	0.229
		*N*	33
	Beck_lately	Correlation coefficient	0.173
		Sig. (2-tailed)	0.320
		*N*	35
	Beck_lifetime	Correlation coefficient	0.145
		Sig. (2-tailed)	0.406
		*N*	35
	IBS_MI	Correlation coefficient	0.341
		Sig. (2-tailed)	0.095
		*N*	25
	IBS_CI	Correlation coefficient	−0.290
		Sig. (2-tailed)	0.159
		*N*	25
	IBS_NPI	Correlation coefficient	−0.260
		Sig. (2-tailed)	0.209
		*N*	25
	Amplitude	Correlation coefficient	1.000
		Sig. (2-tailed)	.
		*N*	39
MDD+NSSI group Spearman's rho	PHQ9	Correlation coefficient	0.403^**^
		Sig. (2-tailed)	0.006
		*N*	45
	Beck_lately	Correlation coefficient	−0.096
		Sig. (2-tailed)	0.527
		*N*	46
	Beck_lifetime	Correlation coefficient	0.108
		Sig. (2-tailed)	0.475
		*N*	46
	IBS_MI	Correlation coefficient	−0.091
		Sig. (2-tailed)	0.594
		*N*	37
	IBS_CI	Correlation coefficient	−0.112
		Sig. (2-tailed)	0.510
		*N*	37
	IBS_NPI	Correlation coefficient	−0.256
		Sig. (2-tailed)	0.126
		*N*	37
	Amplitude	Correlation coefficient	1.000
		Sig. (2-tailed)	.
		*N*	48
MDD+SA group Spearman's rho	PHQ9	Correlation coefficient	0.049
		Sig. (2-tailed)	0.836
		*N*	20
	Beck_lately	Correlation coefficient	0.425
		Sig. (2-tailed)	0.062
		*N*	20
	Beck_lifetime	Correlation coefficient	0.559^*^
		Sig. (2-tailed)	0.010
		*N*	20
	IBS_MI	Correlation coefficient	−0.055
		Sig. (2-tailed)	0.818
		*N*	20
	IBS_CI	Correlation coefficient	−0.382
		Sig. (2-tailed)	0.097
		*N*	20
	IBS_NPI	Correlation coefficient	−0.167
		Sig. (2-tailed)	0.483
		*N*	20
	Amplitude	Correlation coefficient	1.000
		Sig. (2-tailed)	.
		*N*	21

^**^Correlation is significant at the 0.01 level (2-tailed).

^*^Correlation is significant at the 0.05 level (2-tailed).

### 3.4. Time-frequency analysis

**Figure 4:**
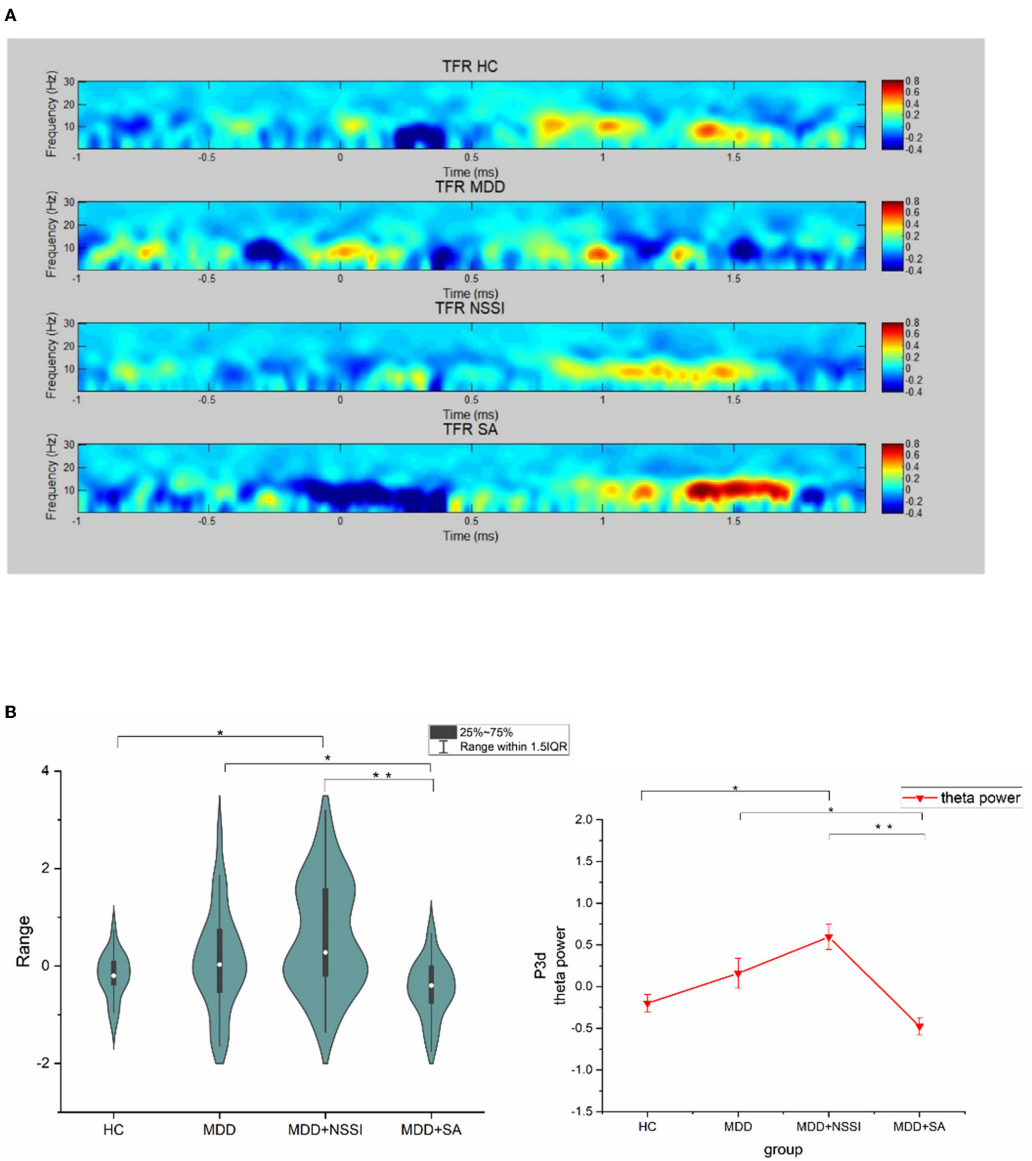
ERSP power (Cz channel) at group level. **(A)** is the power difference (self-injury - neutral cue) at all-time points in each epoch at 0-30Hz, **(B)** is the mean power plot at the time range of P300 component and theta band, *p < 0.01, **p < 0.001.

The current time-frequency analyses were computed using short-term FFTs (MATLAB2013b eeglab toolbox). After baseline correction, the difference between two cues (self-injury-neutral) on the Cz channel for the four groups (HC, MDD, MDD+NSSI, and MDD+SA) is plotted in Figure 4A. The event-related spectral perturbation (ERSP) lasts for a period and induces oscillations (power value) that reflect important information regarding cognitive processes. Thus, we averaged the power value of the P3 time domain (250–450 ms) and frequency domain (5–7 Hz) of interest for statistical analysis. As shown in Table 5, during the P3 period there was a significant difference among the four groups [*F*_(4, 154)_ = 9.818, *p* < 0.001, $\eta_{\text{p}}^{2}$ = 0.18]. As expected, the theta band power showed an upward trend from the HC group through the MDD group to the MDD+NSSI group and there was a significant difference between the HC group and the MDD+NSSI group

**Table 5:** One-way ANOVA of P3d oscillating power at theta band of each group.

**Bonferroni**					
**Dependent variable**	**(I) group**	**(J) group**	**Mean difference (I-J)**	**Std. error**	**Sig**.
P300 theta	HC	MDD	−0.35730	0.25742	1.000
		NSSI	−0.79454^*^	0.23922	0.007
		SA	0.27740	0.26357	1.000
	MDD	HC	0.35730	0.25742	1.000
		NSSI	−0.43725	0.20165	0.191
		SA	0.63470^*^	0.23001	0.039
	NSSI	HC	0.79454^*^	0.23922	0.007
		MDD	0.43725	0.20165	0.191
		SA	1.07195^*^	0.20945	0.000
	SA	HC	−0.27740	0.26357	1.000
		MDD	−0.63470^*^	0.23001	0.039
		NSSI	−1.07195^*^	0.20945	0.000

* *p* < 0.05.

(Bonferroni-adjusted *p* = 0.007). However, there was an obvious turning point between the NSSI group and the SA group. The theta energy of the SA group was lower than that of the MDD group, that is, there was a cliff decline from NSSI to SA, and there were significant differences between the SA group and NSSI group, and MDD group (Bonferroni correction, SA vs. NSSI, *p* < 0.001 vs. MDD, *p* = 0.039), as shown in Figure 4B.

## 4. Discussion

The primary goal of this study was to explore the behavioral development spectrum from MDD through NSSI to suicide. As self-injury behaviors share impulsivity as the common risk, the present study conducted an objective investigation of BIC impairment, expecting to find neuro-electrophysiological indicators to reflect the difference between adolescents with and without self-injury in the context of depression. We found the specificity of BIC impairment in depressive adolescents with a history of suicidal behavior. Specifically, the MDD and MDD+NSSI groups exhibited higher power values than the HC group, showing a gradual upward trend. The trend turned between the MDD+NSSI group and the MDD+SA group, the MDD+NSSI group showed the highest power value among the four groups, and the SA group showed the lowest among the four groups (Figure 4B). These differences suggest that participants in the MDD+SA and MDD+NSSI groups both had deficits in their ability to inhibit the impulsivity of self-injury cues (45), but the different emotional responses to the self-injury cues. Regarding the theta rhythm continually enhancing emotional distress, the different power values may further suggest that the self-injury cue brings emotional distress to the MDD+NSSI group but alleviates negative emotions for the MDD+SA group instead. Defective inhibitory processes lead to impulsive behavior, so in addition to neurodevelopmental diseases, the underlying causes of obsessive–compulsive disorder (OCD) and addiction are closely related to deficits in response-inhibitory control but they differ in the cognitive processes involved in the behavior inhibitory control. The results of functional imaging suggest that in patients with OCD, the combination of an overactive error processing mechanism and response inhibition disorder may be the basis of obsessive–compulsive behavior in disinhibited cues (23). When addicted patients face relevant cues, they are more inclined to the immediate gratification of strong desires in the motor response paradigm due to positive emotional stimulation and immediate desire gratification impulses (31). Findings above are consistent with the difference between NSSI and SA in this study. That is, for the MDD+NSSI group, the self-injury cues are more familiar to obsession, while for the MDD+SA group, the self-injury cues are familiar to addiction. In the presence of depression, the conclusion showed the potential to differentiate individuals who have attempted suicide from those who engaged in repeated self-injury behaviors.

We should also highlight that the participants included in the MDD+SA group had complete suicidal behavior within the last year; this time criterion is longer than the meta-analyses suggested (46). Although the between-group difference in P3d amplitude and theta power in the two-choice oddball task still requires replication and validation of longitudinal data, the findings suggest that, to a certain extent, neuro-electrophysiological indicators could extend the warning time of suicidality. Although any conclusion must remain tentative until a study with a larger sample confirms the reliability and a longitudinal study has confirmed its specificity, results from the present study highlight that time-frequency analyses might contribute to the recognition of suicide attempters in a specific way. Several previous studies have also demonstrated a vital link between psychopathology and the inhibitory control of theta oscillatory dynamics (47–49).

Although we did not find a significant difference between the MDD+NSSI and MDD+SA groups in the P3d amplitude, the NSSI group showed the most considerable amplitude differentiation among individuals without self-injury behavior. This suggests that depressive adolescents with repeated NSSI must expend tremendous effort to inhibit impulsive responses to self-injury cues. Nonetheless, this more significant effort did not act on faster responses (the main effect of the group was not effective in the P3d latencies). Although the results of previous studies of the two-choice oddball paradigm used on BIC impairment are mixed, the results of the present study were consistent with those of our previous study (50) and further, complementary to prior results, suggested that a larger amplitude may not simply be more salient to individuals addicted to self-injury behavior but those with compulsive self-injury behavior. As previous research found that P3 amplitude was negatively associated with no-go errors (51), we consider this greater effort to achieve the accuracy required by the study according to the compensatory mechanism (52), subsequently resulting in a larger amplitude of P3d. The time-frequency analyses of the MDD+NSSI group showed the highest power value, suggesting that the self-injury cue did cause emotional distress to the participants. Indeed, researchers have stated that the conceptual overlap between NSSI and obsessive–compulsive-related disorder (53) illustrates that the MDD+NSSI group did have BIC impairment when exposed to self-injury cues. Considering that participants in the MDD+NSSI group repetitively acted on NSSI behaviors on five or more days in the past year and the motivation was almost entirely to help regulate negative emotions, NSSI behaviors may be more related to obsessive–compulsive disorder than addiction.

In the present study, no significant group differences were found in self-reported levels of impulsivity or the correlation between self-reported impulsivity and P3d amplitude. In both the NSSI and SA studies, self-reported impulsivity seemed unreliable. A meta-analysis demonstrated that a large pooled effect size was evident only when the suicide attempt occurred within a month of behavioral impulsivity assessment. Even in the last month, highly behavioral impulsivity was less likely to make deliberate attempts (46). However, another longitudinal cohort study of children at risk for neurodevelopmental disorders in mid-adolescence suggested that impulsivity and inattention may be particularly important in understanding the onset of NSSI and suicidal behaviors (54). Specifically, adolescents are in the developmental stage of self-cognitive ability whose self-reported results vary significantly. Furthermore, it might not be easy for them, particularly those with a current depression diagnosis, to estimate their impulsivity level objectively and accurately. Interestingly, we found no significant difference among the three depressive groups in the PHQ-9 score, but the correlation analysis showed a significant positive correlation between the P3d amplitude and the PHQ-9 score in the MDD+NSSI group. It suggested that, in addition to the severity of BIC impairment, P3d amplitude also reflects the severity of depression among the NSSI individuals. The results indicated that neuro-electrophysiological indicators might be better and more objective in evaluating depression severity.

The present study achieves considerable homogeneity. In particular, the observation population in the experimental design might improve reproducibility; additionally, it is an indicator of self-injury and suicidality. Most previous suicidality studies have focused on suicidal ideation and suicide attempters compared with non-suicide populations. The commonly used binary classification did not adequately combine suicidality with high-risk factors, such as emotional dysregulation and maladaptive behaviors. Increasing evidence suggests that the onset of suicidal behavior has a long developmental process. As epidemiological surveys and meta-analyses declare, there is above 10 times the significant risk of suicidal behavior among self-injurers.

Previous studies report that multiple episodes of self-harm and psychiatric disorder are highly associated with an increased risk of suicide (10, 20, 55). The present study is one of the few to focus on the spectrum developed from repeated NSSI to suicidal behavior. Furthermore, the employment of ERP or ERSP enabled us to aim at the target cognitive function (i.e., behavior inhibitory control impairment and impulsivity). Additionally, we were able to precisely examine the target component and frequency oscillation of BIC afforded by high temporal resolution. To the best of our knowledge, the present study is the first to objectively evaluate the distinction and correlation between NSSI and suicidal behavior. Moreover, all three depressive groups (MDD, MDD+NSSI, and MDD+SA) had similar depressive symptom levels, indicating a more robust specificity of BIC impairment to self-injury and suicide behavior history.

This study had some limitations. First, the sample size was relatively small, particularly in the MDD+SA group. Consequently, after some of the participants refused the real name of the scale, the correlation between self-reported score and ERP or ERSP index might appear to be a false negative. Second, because of the cross-sectional design of this study, whether the difference between NSSI and SA could predict the onset of subsequent suicidal behavior still requires larger samples for longitudinal studies. This is the next step in our ongoing research exploring suicidality. Third, examining all the related BIC impairment aspects within ERP or ERPS alone was challenging. Due to the poor spatial resolution of the EEG and ERP signal, it is challenging to recognize accurately the brain regions that may respond to the electrophysiological phenomena found in this study. It will be crucial for future research to be performed with neuroimaging data (e.g., fMRI or MEG) to further the understanding of the neuropathophysiological forming processes of suicidality. Finally, NSSI behaviors vary widely in behavioral patterns and severity, which is a major cause of heterogeneity in prediction studies of suicide. There is a strong association between the severity of self-injury and the onset of subsequent suicidal behavior, it is not rigorous enough to discuss NSSI behavior in general. Further studies must be conducted to investigate BIC impairment in different motivations, forms, and severity of self-injury.

## 5. Conclusion

Our study provided neuro-electrophysiological evidence that adolescents with depression involved in self-injury have impairment in BIC when exposed to self-injury cues. Furthermore, the difference in the self-injury between NSSI and suicide might be a warning marker predicting subsequent suicidal behavior in depressive patients combined with the NSSI population, and the warning time may extend longer. Our follow-up research will increase the sample volume for a longitudinal study to verify and replicate our results.

## Data availability statement

The original contributions presented in the study are included in the article/supplementary material, further inquiries can be directed to the corresponding author.

## Ethics statement

The studies involving human participants were reviewed and approved by University Town Hospital of Chongqing Medical University. Written informed consent to participate in this study was provided by the participants' legal guardian/next of kin.

## Author contributions

LM and D-DZ were involved in the study design. LM, LZ, JH, ZJ, XP, XH, SH, and WW were involved in the data collection and scale interviews. LM, D-DZ, and JH were involved in data and statistical analyses. LM wrote the manuscript. LK was involved in study supervision and the manuscript's senior editor. All authors had full access to all data in the study. Furthermore, they all take responsibility for the data's integrity and the data analysis's accuracy. All authors contributed to the article and approved the submitted version.
